# Lyme Carditis in an Immunocompromised Patient

**DOI:** 10.1155/2013/380734

**Published:** 2013-08-01

**Authors:** Matthew F. Ryan, Coben Thorn

**Affiliations:** Department of Emergency Medicine, University of Florida, 1329 SW 16th Street, P.O. Box 1000186, Gainesville, FL 32610-0186, USA

## Abstract

We present a case of a 68-year-old man with a history of liver transplant and of chronic immunosuppression therapy who presented to the emergency department (ED) for fevers and worsening fatigue for two days. On further investigation, the patient was found to have a new first-degree heart block on his electrocardiograph. Coupled with the history of a recent tick bite, the patient was diagnosed with vector-borne carditis. Although the patient's titers for various vectors remained negative, due to a long history of immunosuppression, he was treated for Lyme disease and his heart block completely resolved with antibiotic treatment. We describe details of the case as well as discuss the impacts of immunosuppression on vector-borne disease. Immunosuppressed patients represent a special population and can present with chief complaints made even more complicated by their medical history, and this case illustrates the importance of being mindful of how immunosuppression can affect a patient's presentation. As the efficacy of antirejection medications improved, the ED may see an increasing number of patients with solid organ transplants. A greater understanding of this special patient population is key to formulating optimal treatment plans.

## 1. Introduction

The number of solid organ transplantations in the United States has increased over the past several decades. Several reasons account for this including better surgical techniques, improved immunosuppressive regiments, and broader use of prophylaxis to prevent infections [1]. Still, one of the greatest barriers to patient survival and graft rejection is infection in this selected patient population secondary to being immunocompromised. Moreover, because of improved longitudinal care, transplant recipients have become more susceptible to a broader array of infections [2]. For the emergency medicine physician, this means encounters with patients with solid organ transplants are becoming more common, consistent with the rise in patient acuity and steadily growing number of patients seeking care in the ED. Furthermore, common infections such as line infections and less common ailments such as opportunistic infections can occur in this patient population which requires the emergency medicine physician to be ever cautious [3]. 

We discuss herein a case involving a patient with a liver transplant on strong immunosuppressants who developed an erythematous rash after a tick bite; subsequently his condition progressed to disseminated illness with a concomitant heart block. Of significance is our patient was indeed immunosuppressed and had an atypical presentation for a vector-borne illness as well as the course of his disease. Based on a high clinical suspicion and evidence of acute disease the patient was treated for Lyme disease resulting in complete resolution of his symptoms. Ultimately, the patient carried the clinical diagnosis of Lyme carditis caused by the spirochete *Borrelia burgdorferi.*

## 2. Case Report 

A 68-year-old male with history of alcoholic liver disease status-post-liver transplant presented to the emergency department (ED) complaining of two days of fevers, diffuses myalgias, and arthralgias and progressively worsening fatigue. The patient denied chest pain, syncope, dizziness, shortness of breath, cold or flu-like symptoms, abdominal pain, dysuria, or focal neurologic deficits. He also noted he had a persistent rash on his right calf where he had removed a tick several weeks ago; he was unsure how long the tick was on him before he noticed it. The patient indicated he was in his normal state of health until 2 days ago and he had no prodromal symptoms. The patient lives in Florida and denied any recent travel. 

The patient's past medical history was significant for type-2 diabetes, hypertension, and liver failure secondary to alcohol abuse resulting in a liver transplant in 2001. For his medical issues, the patient took Gemfibrozil, Glipizide, Lisinopril, Metolazone, Paricalcitol, Simvastatin, and Tacrolimus. The patient had an allergy to penicillin (hives). The patient has abstained from alcohol and tobacco since his transplant.

The patients had a pulse in the 90s, but otherwise his vital signs were unremarkable. The patient was ill appearing but nontoxic and not in any distress. He complained of intense pain in all extremities and in his joints, back, and neck with both passive and active movements. The skin exam revealed a ca. 2 cm ovoid erythematous macular rash on his posterior right calf which was nonblanchable, normal temperature to the touch, showed no signs of overriding cellulitis, and was neither painful nor pruritic. The rash had no obvious central clearing which would be consistent with erythema migrans (EM). The remainder of the patient's physical exam was normal.

**Figure 1:**
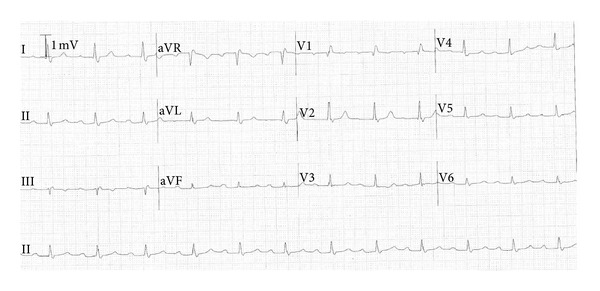
Patient's EKG demonstrating new onset 1st degree heart block with PR interval of ca. 300 ms.

Blood work including Lyme and Ehrlichiosis titers was drawn, and an electrocardiogram (EKG, shown in Figure 1) was ordered. The EKG showed a new first-degree heart block with PR intervals of approximately 300 ms which was not seen on his older EKGs. Previous EKGs for the patient demonstrated normal intervals.

During his ED visit, the patient requested medication for his severe muscle and joint pain and was given 4 mg of morphine intravenously (IV). However, approximately 10 minutes after receiving the morphine the patient's mental status declined and he was minimally responsive; the cardiac monitor showed a brief run of sinus bradycardia in the 20s with a type II heart block and a drop in his systolic blood. He was given 0.5 mg of atropine IV as well as a one-liter normal saline IV fluid bolus to which he rapidly responded and his vital signs and mental status normalized. 

The blood work showed the patient to be thrombocytopenic, and the balance of his laboratory studies including his liver panel was within normal limits. A peripheral smear was negative for intracytoplasmic inclusions (morulae) indicative of Ehrlichia infections.

Based on the patient's history, physical exam, and noted EKG changes, the patient was started on IV ceftriaxone and oral doxycycline for possible Lyme carditis, and he was admitted to the cardiology service for continued care. While an inpatient, the patient developed a right-bundle-branch block (RBBB). Over the next few days on antibiotics the patient's PR interval returned to normal from a high of 400 ms and his RBBB completely resolved. The patient was continued on doxycycline for 14 days after discharge. Of note, our patient's Lyme and Ehrlichiosis titers remained normal. Later follow-up showed his EKG remains normal, and the patient had no long-term symptoms.

## 3. Discussion

Lyme carditis typically occurs during the early disseminated phase (weeks to months) after a *Borrelia burgdorferi* infection [3, 4]. Cardiac manifestations occur in up to 10% of patients, the most common of which is AV block (our patient had a new onset 1st degree heart block). Complete heart block (requiring a pace maker) and dilated cardiomyopathy can occur at unpredictable rates [1]. The diagnosis can be made in the presence of disseminated symptoms of Lyme disease with new onset EKG changes [2]. This was the case for our patient who presented with diffuse myalgias and arthralgias in all major joints with associated malaise (representing disseminated systemic disease) and significant EKG changes. Because of the history of a recent tick bite concomitant with symptoms consistent with disseminated Lyme disease, he was empirically started on antibiotics. Confounding the diagnosis was the fact our patient was on immunosuppressants for his liver transplant which likely explains his failure to seroconvert, a similar situation for other immunocompromised patients [5, 6].

The patient was unsure how long the tick was on his leg before he removed it, and thus his rash may have represented erythema migrans; about 20% of immunocompetent patients with Lyme disease fail to develop a rash typical of erythema migrans, and this number is postulated to be higher in immunocompromised patients [5, 6]. For example, Maraspin and coworkers noted a small cohort of patients with solid-organ transplants who developed EM failed to seroconvert [5]. This underscores the limited use of serological testing in the immunocompromised patient population including similar populations on immunosuppression for treatment of autoimmune or rheumatologic disorders, for example, and those who are immunocompromised secondary to infection (say, HIV) [7]. Therefore, clinical criteria are needed and recommended [2] to diagnose both erythema migrans and the manifestations of disseminated Lyme disease or other tick-borne or vector-borne diseases. In this case the diagnosis was made based on clinical suspicion, significant EKG changes along with their acute manifestations (symptomatic bradycardia), and the patient's history of a tick bite. 

## 4. Conclusion 

There are several points worth noting as follows. First, solid organ transplantation is becoming more successful due to improved efficacy of antirejection medications; thus the prevalence of patients with organ transplants will likely increase leading to an increase in these patients seeking care for unrelated issues. Next, because the efficacy of antirejection regiments has improved in recent years, patients with solid organ transplants as well other conditions may present with common medical conditions but with atypical symptoms. Finally, EDs will continue to see an increasing number of patients with increasingly complex medical conditions. Exposure and understanding of at-risk patient populations are requisite of emergency medicine providers but, nevertheless, and by assimilating fundamental knowledge of commonly encountered pathologies, we can draw logical conclusions even about seemingly rare and unlikely diagnosis.
